# Design, Synthesis and Cytotoxic Evaluation of Novel Chalcone Derivatives Bearing Triazolo[4,3-*a*]-quinoxaline Moieties as Potent Anticancer Agents with Dual EGFR Kinase and Tubulin Polymerization Inhibitory Effects

**DOI:** 10.3390/molecules23010048

**Published:** 2017-12-25

**Authors:** Mohamed Alswah, Ashraf H. Bayoumi, Kamal Elgamal, Ahmed Elmorsy, Saleh Ihmaid, Hany E. A. Ahmed

**Affiliations:** 1 Pharmaceutical Organic Chemistry Department, Faculty of Pharmacy, Al-Azhar University, Cairo, Nasr City 11884, Egypt; drmohammedalswah@yahoo.com (M.A.); bayoumi.ashraf@hotmail.com (A.H.B.); drkamalelgaml72@gmail.com (K.E.); ahmedelmorsy232@yahoo.com (A.E.); 2 Pharmaceutical Organic Chemistry Department, Faculty of Pharmacy, Delta University for Science and Technology, Mansoura 11152, Egypt; 3 Pharmacognosy and Pharmaceutical Chemistry Department, Pharmacy College, Taibah University, Al-Madinah Al-Munawarah 41477, Saudi Arabia; saleh_ihmaid@yahoo.com.au

**Keywords:** triazolo[4,3-*a*]quinoxaline, chalcone, EGFR, molecular modeling, anticancer, docking

## Abstract

A series of hybrid of triazoloquinoxaline-chalcone derivatives **7a**–**k** were designed, synthesized, fully characterized, and evaluated for their cytotoxic activity against three target cell lines: human breast adenocarcinoma (MCF-7), human colon carcinoma (HCT-116), and human hepatocellular carcinoma (HEPG-2). The preliminary results showed that some of these chalcones like **7b**–**c**, and **7e**–**g** exhibited significant antiproliferative effects against most of the cell lines, with selective or non-selective behavior, indicated by IC_50_ values in the 1.65 to 34.28 µM range. In order to investigate the mechanistic aspects of these active compounds, EGFR TK and tubulin inhibitory activities were measured as further biological assays. The EGFR TK assay results revealed that the derivatives **7a**–**c**, **7e**, and **7g** could inhibit the EGFR TK in the submicromolar range (0.093 to 0.661 µM). Moreover, an antitubulin polymerization effect was noted for the active derivatives compared to the reference drug colchicine, with compounds **7e** and **7g** displaying 14.7 and 8.4 micromolar activity, respectively. Furthermore, a molecular docking study was carried out to explain the observed effects and the binding modes of these chalcones with the EGFR TK and tubulin targets.

## 1. Introduction

Cancer ranks second of the top ten mortal diseases around the world, especially in the industrialized countries, highlighting the imperative need to discover novel therapies and approaches to cure this disease [1,2]. Nowadays, anticancer chemotherapy is still the main method applied in the treatment of cancer. In spite the development of new anticancer agents, the accumulation of toxicity often limits their application as antitumor drugs [3,4]. As a result, there is an urgent need to discover and develop new classes of anticancer drugs to combat the growth of drug resistance [5]. Our literature survey revealed that quinoxalines and their fused analogs are attractive candidates in medicinal chemistry as they constitute the building blocks of a wide range of compounds possessing a numerous interesting biological properties such as anticancer [6,7], antihistaminic [8], antiviral [9], antimicrobial [10,11,12], antifungal [13] antitubercular [14,15] and anti-inflammatory effects [16]. Among the biologically active pharmacophores, amides have been associated with fascinating anticancer [17], antitubercular [18,19], anti-inflammatory [20] and imines are reported to have analgesic [21] properties. In addition, they have shown anti-protozoal [22], antidepressant [23], anticonvulsant [24] and kinase inhibitor properties [25,26]. Moreover, a series of fused 1,2,4- and 1,2,3-triazoles were reported as interesting molecules with dual fluorescence effects [27,28,29]. In addition, 1,2,4-triazolo[4,3-*a*]quinoxalines bearing a triazole moiety have been reported as promising anticancer agent motifs [30,31,32,33,34,35,36,37,38,39]. Their mechanism of action apparently involves binding to DNA where they function as intercalating agents [40,41]. Chalcones (1,3-diaryl-2-propen-1-ones) are an interesting scaffold in which the two aromatic rings are connected by a three-carbon α,β-unsaturated carbonyl system. Chalcone derivatives with diverse chemical architectures are quite significant in anticancer drug discovery and hence are in the center of attention of drug hunters [42]. The anticancer activity of chalcones might be due to molecular alterations such as induction of apoptosis, DNA and mitochondrial damage, inhibition of angiogenesis, tubulin inhibition, kinase inhibition, and also drug efflux protein activities [43,44].

Recently, the tubulin inhibitory potential of boronic acid chalcone analogs [45], cinnamic acyl sulfonamide derivatives [46] and chalcone-based azacarbolines [47] has been reported. In view of these facts, we herein report a facile synthesis of new series of triazoloquinoxaline-chalcone derivatives as a privileged bioactive scaffold. The presence of the triazoloquinoxaline moiety linked to the aromatic chalcone scaffold moiety might provide a rigid arrangement of the two aryl rings that enhances their activity. This design strategy led to a set of novel compounds that might have cytotoxic effects via EGFR TK and tubulin inhibitory mechanisms and looks like the reference drugs.

## 2. Rationale Study

Several chalcones have been reported to act as cytotoxic agents or as microtubule destabilizing agents, targeting the colchicine binding site [48,49]. The majority of these are naturally occurring compounds substituted with electron donating hydroxy and/or methoxy groups at various positions [50,51]. It was reported that the [1,2,4]triazolo[4,3-*a*]quinoxaline moiety was introduced as bromodomain and extra-terminal (BET) protein inhibitors for cancer treatment [52].

**Figure 1:**
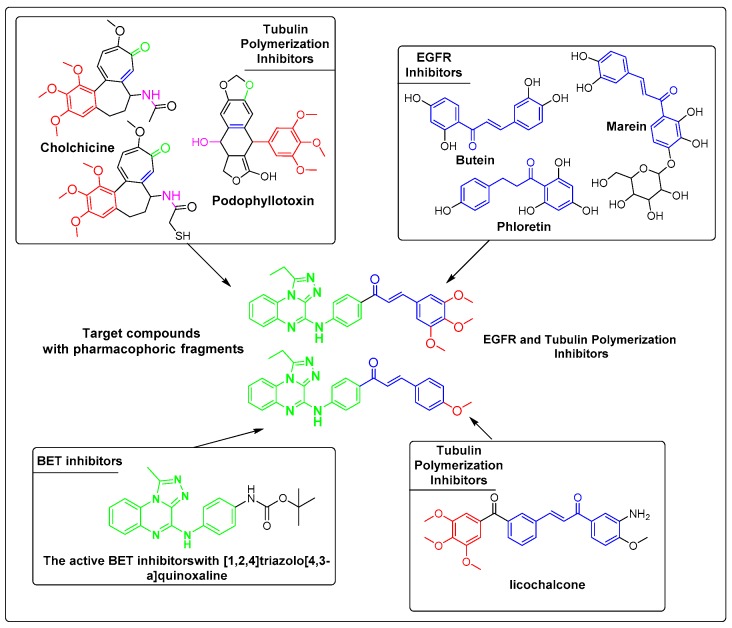
Reference compound sets with different structures and mechanisms. The highlighted substructures reveal the pharmacophore points related to the reported biological effect.

To establish more advanced structure–activity relationships around chalcones, the synthesis of compounds with more diverse substitution patterns is of great importance. Our research is aimed at the development of novel scaffold-based chalcones with incorporation of [1,2,4]triazolo[4,3-*a*]quinoxalines as essential fragments for the design of anticancer agents [49]. In the present work, we unveil the discovery of a novel scaffold with [1,2,4]triazolo[4,3-*a*]quinoxaline conjugated with chalcones as dual EGFR and tubulin polymerization inhibitors potentially useful in cancer treatment (Figure 1).

## 3. Results and Discussion

### 3.1. Chemistry

**Scheme 1:**
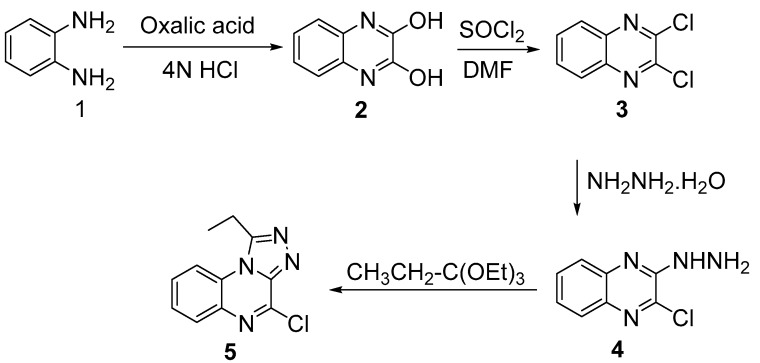
Synthesis of *N*-aryl and heteroaryl triazoloquinoxalines.

The precursor chlorotriazoloquinoxaline **5** was obtained after multiple reaction steps starting with condensation of *o*-phenylenediamine (**1**) with oxalic acid, followed by a nucleophilic substitution reaction affording dichloroquinoxaline compound **3**. Hydrazinolysis of this compound produced the intermediate compound **4**. Cyclization of compound **4** with excess triethylortho-propionate gave the corresponding fused chlorotriazoloquinoxaline **5** in good yield (Scheme 1).

**Scheme 2:**
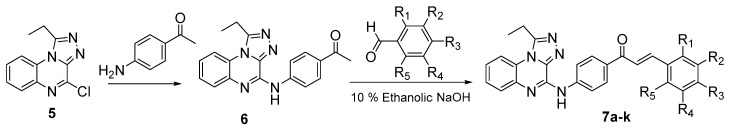
Synthesis and structures of triazoloquinoxaline chalcone derivatives **7a**–**k**.

Analytically pure triazoloquinoxaline **5** was obtained by recrystallisation from an appropriate solvent (see Experimental). Its structural proof was based on a correct elemental analyses as well as on IR and NMR spectral data. The title compound **6** was next prepared by dissolving 4-chloro-1-ethyl[1,2,4]triazolo[4,3-*a*]quinoxaline (**5**) freely in acetonitrile and heated under reflux with different heteroaromatic or aromatic amines and a catalytic amount of TEA to produce a nucleophilic substitution of the Cl atom giving an intermediate that was trapped by highly electronegative nitrogen atoms and yielded the corresponding *N*-aryl substituted derivatives in a short time (4–6 h) with high purity (Scheme 2). The structure of compounds **6** was confirmed by elemental analysis and spectral data.

Substituted triazoloquinoxaline chalcone derivatives **7a**–**k** were finally prepared by condensation of aromatic aldehydes with compound **6**. The structures of these novel compounds were confirmed by their elemental analyses and spectral data. The characteristic IR band of the (C=O) group appears at 1734 cm^−1^. In addition, ^1^H-NMR revealed that the characteristic doublet signals of the olefinic protons (CH=CH) occur between δ 7.4–8.4 ppm.

### 3.2. Cytotoxicity Screening

The newly synthesized chalcone compounds **7a**–**k** were evaluated for their in vitro anticancer activity against three cancer cell lines—HCT-116, MCF-7 and HepG-2—according to the MTT assay method using doxorubicin as reference drug. The in vitro anticancer activity results reveal that some of the chalcone compounds exhibited excellent activity against these tumor cells. The compounds **7b**, **7e** and **7g** bearing mono- or trimethoxy-substituted benzene rings showed moderate to good anticancer activity with IC_50_ values of 1.65 to 34.28 µM against the three cell lines and were mostly not selective towards any particular cell line.

Substitution of the electron-attracting groups as in the chloro and nitro derivatives **7h**–**k** resulted in no significant anticancer activity, as indicated by their IC_50s_ more higher than 100 µM, whereas the introduction of electron-releasing groups such as methyl (compound **7d**) produced low activity while substitution with hydroxy groups in 4-position on the benzene ring (compounds **7c** and **7f**) enhanced the anticancer activity remarkably, selectively or non-selectively. Compound **7a** without substitution did not show significant activity against any of the tested cell lines. These results suggest that the substitution patterns on the terminal aromatic ring of the chalcones plays a vital role in the modulation of cytotoxicity. The results are summarized in Table 1.

### 3.3. EGFR Inhibitory Assay

As the designed compounds had good antiproliferative activity, in vitro EGFR inhibition capability was firstly evaluated for the target cytotoxic derivatives to see whether their cytotoxicity was mediated by kinase inhibition. As shown in Table 2, the compounds **7a**–**c**, **7e**, and **7g** showed excellent potent EGFR inhibition activity comparable or better than that of the reference drug staurosporine. Derivative **7g** exhibited the most potent activity against the kinase target which was consistent with its cytotoxic effect. In addition, compounds **7b** and **7c** showed good inhibitory activity with an IC_50_ range of 0.127 to 0.136 μM. Moreover, chalcone **7e** displayed promising inhibitory effect, indicated by an IC_50_ of n0.083 against EGFR at 10 μM. Overall, most of the chalcone derivatives showed potent EGFR TK inhibitory activity and deserve further studies and optimization as anticancer agents.

### 3.4. Tubulin Assay

Tubulin is the principal component of the eukaryotic cytoskeleton and participates in several crucial cellular processes, including cell replication. As evident from the literature, chalcones show cytotoxic activity through the inhibition of tubulin polymerization, hence impeding microtubule formation [48]. Compounds **7d**, **7f**, and **7h**–**k** revealed no significant influence on tubulin assembly, which suggests a different mechanism for the observed cytotoxicity than tubulin inhibition (Table 2). However, five compounds (**7a**–**c**, **7e**, and **7g**) showed significant inhibition of tubulin assembly. As reported in Table 2, **7e** and **7g** efficiently inhibited tubulin polymerization with IC_50_ values of 14.7 and 8.84 μM, respectively. These results suggest that the molecular target of these chalcone derivatives might be tubulin, and their strong anti-tubulin polymerization activity corresponds well with their cytotoxicity.

### 3.5. Molecular Docking Analyses

**Figure 2:**
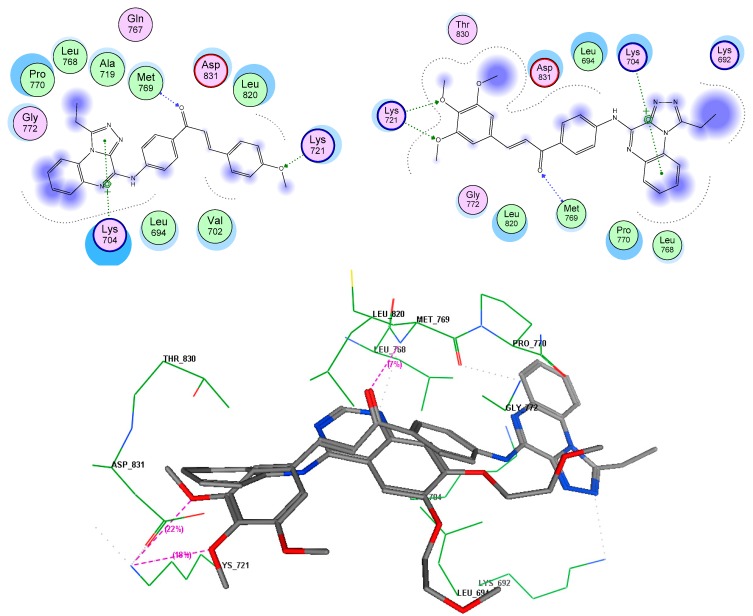
Molecular interaction graph of target compounds **7e** and **7g** in the EGFR TK ATP pocket.

**Figure 3:**
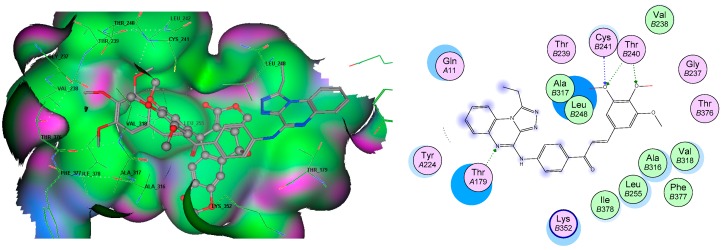
Molecular interactions graph of target compounds **7g** in the tubulin pocket.

To rationalize the experimental results obtained, molecular docking studies were performed on two representative potent compounds (**7e** and **7g**) against the two interesting targets, EGFR TK and tubulin by a method similar to a previously reported successful approach [53]. According to the interaction results presented in Table 3, two compound poses showed high binding energies with EGFR and tubulin targets, with energie s ranging from −12.8 to −15.5 kcal/mol. All compounds formed a network of molecular interactions (H-bonds, vdw, and π-aromatic) with the active site residues of EGFR when analyzed in 2D plots as shown in Figure 2. Compound **7e** formed stable hydrogen bonds with the terminal methoxy groups and linker carbonyl fragments through the Lys721 and Met769 residues. In addition, the role of triazoloquinoxaline in stabilizing the compound inside the ATP binding pocket through the aromatic hydrophobic interaction in hydrophobic pocket produced by the Lys704 residue. The same behavior was exhibited in case of compound **7g** by forming various interactions in the ATP pocket. It is well established that the colchicine binding site is generally present at the interface of α,β-tubulin heterodimers. The docking arrangement of active anti-tubulin **7g** compound fit well into the colchicine binding pocket present at the α and β interfaces of tubulin with the lowest binding energy. The chalcone part occupied the hydrophobic pocket formed of Leu248, Ala315, Ile378, Leu255 residues present in the β-subunit, whereas the quinoxaline ring protruded towards the α subunit with the help of hydrogen bonding to the Thr179 residue (Figure 3).

### 3.6. SAR Analyses

The SAR study revealed several crucial structural requirements which enhanced the potency of these chalcone compounds, **7a**–**c**, **7e**, and **7g**. Incorporation of the triazoloquinoxaline moiety into an active scaffold like chalcone with reported anticancer activity resulted in enhanced potency. This explains the importance of the molecular hybridization of active fragments.

**Figure 4:**
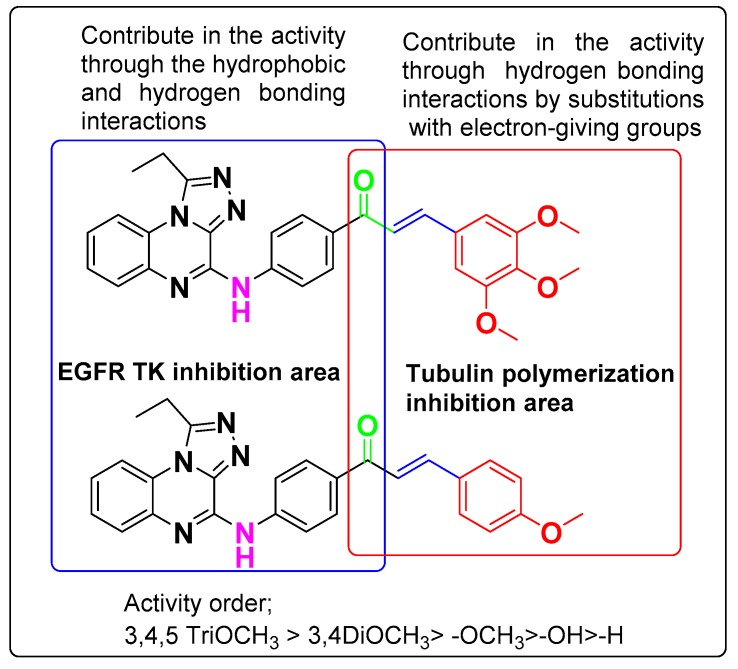
Structure activity relationship of chalcone derivatives **7e** and **7g**.

As shown in the activity and modeling reports, the quinoxaline ring plays a crucial role in the EGFR inhibitory effect, whereas in the case of tubulin it increases the stability of the structure in the colchicine bind site. Moreover, the terminal aromatic ring in chalcone is essential for tubulin inhibition and a suitable substitution pattern is a must. All compounds with OH or OCH_3_ groups exhibited anti-EGFR and tubulin polymerization effects. It is noteworthy to observe that substituents like OCH_3_ at the 3th and 4th positions have a significant impact on the activities (Figure 4). Finally, molecular hybrids of chalcone and triazoloquinoxaline fragments afforded potent lead compounds while preserving the pharmacophore points essential for EGFR and tubulin polymerization inhibition.

## 4. Experimental Section

### 4.1. General Information

All melting points were taken on a LA 9000 SERIES digital melting point apparatus (Electrothermal, Staffordshire, UK) and are uncorrected. IR spectra were recorded on a SP 1000 IR spectrophotometer (Pye Unicam, Cambridge, UK). ^1^H-NMR and ^13^C-NMR spectra were recorded on an AC 300 MHz NMR spectrometer (Bruker, Billerica, MA, USA) at 300 for ^1^H and 100 MHz for ^13^C, respectively. All ^1^H- and ^13^C-NMR spectral results are recorded as chemical shift (δ) values. Chemical shifts recorded in DMSO-*d*_6_ are relative to the solvent peak of 2.5 ppm for ^1^H-NMR spectra and 39.5 ppm for ^13^C-NMR spectra. Mass spectra were recorded on a 5988 spectrometer (Hewlett Packard, Palo Alto, CA, USA). Microanalyses were carried out at the Cairo University Microanalytical Center. Progress of the reactions was monitored by TLC using TLC sheets precoated with UV fluorescent silica gel (Merck 60 F254 plates, Merck & Co., Kenilworth, NJ, USA) and different solvents as mobile phases and visualized using UV lamp. Antimicrobial and antitumor activities were evaluated at the Regional Center for Mycology and Biotechnology at Al-Azhar University, Cairo, Egypt.

### 4.2. Chemistry

#### 4.2.1. Synthesis of 2,3-(1*H*,4*H*)-Quinoxalinedione (**2**)

A solution of oxalic acid (27.1 g, 0.215 mol) in 4 N aqueous HCl (50 mL) was added to a solution of *o*-phenylenediamine (20.9 g, 0.193 mol) in 4 N HCl (150 mL), and the resulting solution was heated under reflux for 2 h. The reaction mixture was cooled to ambient temperature, and the resulting precipitate was isolated by filtration, washed with water, dried, recrystallized from ethanol giving 30.5 g of **2** as dark red microcrystalline solid (30.5 g, 98%); m.p. > 360 [54].

#### 4.2.2. Synthesis of 2,3-Dichloroquinoxaline (**3**)

DMF (0.045 g, 0.00062 mol) was added dropwise to a slurry of 2,3-dihydroxyquinoxaline (**2**, 2.0 g, 0.012 mol) and thionyl chloride (3.7 g, 0.031 mol) in 1,2-dichloroethane (20 mL). The resulting reaction mixture was heated to reflux for 2 h then concentrated to dryness. The residue was dissolved in 1,2-dichloroethane (25 mL) and concentrated to dryness. The resulting solid was crystallized from CH_3_CN/H_2_O, giving 2.3 g (95%) of **3** as fine, off-white needles. m.p. 148–150 °C [55].

#### 4.2.3. Synthesis of 2-Chloro-3-hydrazinylquinoxaline (**4**)

A mixture of (10.0 g, 0.1 mol) of 2,3-dichloroquinoxaline and (10.0 g, 0.22 mol) of hydrazine hydrate in 200 mL of ethanol was stirred for 16 h at 25 °C. The resulting precipitate was filtered, and the solid was washed with ethanol and air dried to give 7.5 g (100%) of crude product as a yellow powder, m.p. 181–182 °C [23].

#### 4.2.4. Synthesis of 4-Chloro-1-ethyl-[1,2,4]triazolo[4,3-*a*]quinoxaline (**5**)

DMF (0.045 g, 0.00062 mol) was added dropwise to a slurry of 1-ethyl-[1,2,4]triazolo[4,3-*a*]quinoxalin-4(5*H*)-one (1.0 g, 0.1 mol) and thionyl chloride (2.77 g, 0.5 mol) in 1,2-dichloroethane (100 mL). The resulting reaction mixture was heated to reflux for 6 h filtered while hot then concentrated to dryness. The precipitate purified by stirring with a mixture of diethyl ether and n-hexane (1:1) then filtered and air dried to give 1.09 g (85%) of product as a light yellow powder. m.p. 158–160 °C [23].

#### 4.2.5. Synthesis of 1-(4-((1-ethyl-[1,2,4]triazolo[4,3-*a*]quinoxalin-4-yl)amino)phenyl)ethan-1-one (**6**)

A reaction mixture of 4-chloro-1-ethyl-[1,2,4]triazolo[4,3-*a*]quinoxaline (**5**, 0.3 g, 0.01 mol) and a corresponding aromatic amine (0.011 mol) in dry CH_3_CN (10 mL) of containing a few drops of TEA was refluxed for 4–6 h. After concentrating the precipitated solid was filtered and crystallized from ethanol affording the desired product **6**. Yield (40%); m.p. 235–237 °C; IR cm^−1^ (KBr); 3412 (N–H), 3060 (C–H aromatic), 2937 (C–H aliphatic), 1629 (C=N); ^1^H-NMR (DMSO-*d*_6_): 9.99 (s, 1H, N–H exchangeable with D_2_O), 7.67–8.30 (m, 8H, Ar-H), 3.43 (q, 2H, *J* = 8, CH_3_CH_2_), 1.49 (t, 3H, *J* = 8, CH_3_CH_2_); ^13^C-NMR (DMSO-*d*_6_) δ 194.4, 161.5, 152.2, 145.1, 142.1, 135.9, 131.0, 129.6, 128.5, 125.9, 124.8, 120.7, 112.5, 111.8, 26.5, 21.5, 12.1; MS (*m*/*z*), 297 (0.97, M + 1), 296 (2.51, M+), 267 (7.53), 212 (2.06), 128 (100), 84 (2.34), 77 (16.39), 76 (78.85); Anal. Calcd. for C_19_H_17_N_5_O: C, 68.87; H, 5.17; N, 21.13, Found: C, 68.85; H, 5.16; N, 21.14.

#### 4.2.6. General Method for the preparation of (*Z*)-{4-[(1-Ethyl-[1,2,4]triazolo[4,3-*a*]quinoxalin-4-yl)amino]phenyl}-3-substituted phenylprop-2-en-1-ones **7a**–**k**

A mixture of the appropriate aromatic aldehyde (0.012 mol) and 1-(4-((1-ethyl-[1,2,4]triazolo[4,3-*a*]quinoxalin-4-yl)amino)phenyl)ethan-1-one (**6**, 0.01 mol) dissolved in ethanol (70 mL) was added slowly to an aqueous solution of potassium hydroxide (0.0128 mol) in water (10 mL). The reaction mixture was stirred in crushed-ice bath for half hour. Stirred at 20–25 °C for 4–6 h. The mixture was filtrated and the solid was washed with cold water and ethanol. The product was crystallized from ethanol to give the corresponding chalcones **7a**–**k**, respectively.

##### *(E)-1-(4-((1-Ethyl-[1,2,4]triazolo[4,3-a]quinoxalin-4-yl)amino)phenyl)-3-phenylprop-2-en-1-one* (**7a**)

Yield (82%); m.p. 260–262 °C; IR cm^−1^ (KBr); 3443 (N–H), 2967 (C–H aromatic), 2922 (C–H aliphatic), 1732 (C=O), 1649 (C=N); ^1^H-NMR (DMSO-*d*_6_): 10.51 (s, 1H, NH exchangeable with D_2_O), 7.41–8.44 (m, 14H, Ar–H + 1H, C–H-arylidene), 3.45 (q, 2H, *J* = 7.5, CH_3_CH_2_), 1.50 (t, 3H, *J* = 7.5, CH_3_CH_2_); ^13^C-NMR (DMSO-*d*_6_) δ 189.7, 161.5, 152.2, 146.7, 145.1, 142.1, 135.9, 135.2, 132.0, 128.7, 128.6, 128.5, 127.9, 125.9, 124.8, 121.3, 120.7, 112.5, 111.8, 21.5, 12.1; MS (*m*/*z*), 420 (3.61, M + 1), 419 (11.65, M+), 239 (39.39), 222 (2.82), 212 (2.19), 197 (1.66), 98 (58.84), 71 (82.65), 57 (100); Anal. Calcd. for C_26_H_21_N_5_O; C, 74.44; H, 5.05; N, 16.70, Found: C, 74.73; H, 5.07; N, 16.75.

##### *(E)-1-(4-((1-Ethyl-[1,2,4]triazolo[4,3-a]quinoxalin-4-yl)amino)phenyl)-3-(2-methoxyphenyl)prop-2-en-1-one* (**7b**)

Yield (37%); m.p. 264–266 °C; IR cm^−1^ (KBr); 3446 (N–H), 3099 (C–H aromatic), 2938 (C–H aliphatic), 1731 (C=O), 1649 (C=N); ^1^H-NMR (DMSO-*d*_6_): 10.54 (s, 1H, N–H exchangeable with D_2_O), 7.04–8.45 (m, 13H, Ar–H, 1H, C–H-arylidene), 3.91 (s, Ar–OCH_3_), 3.45 (q, 2H, *J* = 7.5, CH_3_CH_2_), 1.49 (t, 3H, *J* = 7.5, CH_3_CH_2_); ^13^C-NMR (DMSO-*d*_6_) δ 189.7, 161.5, 159.5, 152.2, 146.7, 142.1, 141.1, 135.2, 132.0, 128.9, 128.7, 127.9, 125.8, 125.7, 124.8, 121.3, 120.9, 120.7, 114.5, 112.5, 111.8, 56.4, 21.5, 12.1; MS (*m*/*z*), 450 (0.89, M + 1), 449 (3.57, M+), 265 (100), 183 (24.52), 128 (62.61), 104 (42.53), 77 (36.27); Anal. Calcd. for C_27_H_23_N_5_O_2_; C, 72.14; H, 5.16; N, 15.58, Found: C, 72.42; H, 5.18; N, 15.64.

##### *(E)-1-(4-((1-Ethyl-[1,2,4]triazolo[4,3-a]quinoxalin-4-yl)amino)phenyl)-3-(2-hydroxyphenyl)prop-2-en-1-one* (**7c**)

Yield (21%); m.p. 261–263 °C; IR cm^−1^ (KBr); 3446 (O–H), 3359 (N–H), 3062 (C–H aromatic), 2933 (C–H aliphatic), 1734 (C=O), 1670 (C=N); ^1^H-NMR (DMSO-*d*_6_): 10.48 (s, 1H, N–H exchangeable with D_2_O), 9.01 (s, 1H, Ar–O–H exchangeable with D_2_O), 7.46–8.39 (m, 13H, Ar–H, 1H, C–H-arylidene), 3.47 (q, 2H, *J* = 7.5, CH_3_CH_2_), 1.51 (t, 3H, *J* = 7.5, CH_3_CH_2_); ^13^C-NMR (DMSO-*d*_6_) δ 189.7, 161.5, 159.5, 152.2, 146.7, 142.1, 141.0, 135.4, 132.2, 129.3, 128.9, 128.7, 127.9, 125.7, 124.8, 122.6, 121.9, 121.3, 120.7, 117.5, 112.7, 111.3, 21.5, 12.1; MS (*m*/*z*), 435 (4.79, M+), 331 (25.09), 234 (12.55), 120 (25.48), 84 (81.50), 77 (8.54), 57 (50.87), 44 (100); Anal. Calcd. for C_26_H_21_N_5_O_2_; C, 71.71; H, 4.86; N, 16.08, Found: C, 71.99; H, 4.84; N, 16.10.

##### *(E)-1-(4-((1-Ethyl-[1,2,4]triazolo[4,3-a]quinoxalin-4-yl)amino)phenyl)-3-(p-tolyl)prop-2-en-1-one* (**7d**)

Yield (43%); m.p. 247–249 °C; IR cm^−1^ (KBr); 3435 (N–H), 3029 (C–H aromatic), 2921 (C–H aliphatic), 1733 (C=O), 1684 (C=N); ^1^H-NMR (DMSO-*d*_6_): 10.53 (s, 1H, N–H exchangeable with D_2_O), 7.26–8.45 (m, 13H, Ar–H, 1H, C–H-arylidene), 3.47 (q, 2H, *J* = 7.5, CH_3_CH_2_), 2.36 (s, Ar–CH_3_), 1.51 (t, 3H, *J* = 7.5, CH_3_CH_2_); ^13^C-NMR (DMSO-*d*_6_) δ 189.7, 161.5, 152.2, 145.7, 142.1, 137.4, 135.4, 132.2, 132.0, 128.9, 128.7, 128.5, 127.9, 125.7, 124.8, 121.3, 120.7, 112.8, 111.3, 21.7, 21.5, 21.3, 12.1; MS (*m*/*z*), 434 (28.46, M + 1), 433 (100, M+), 212 (3.38), 432 (59.57), 233 (30.53), 212 (6.74), 145 (26.28), 128 (3.35), 115 (46.99), 77 (7.26); Anal. Calcd. for C_27_H_23_N_5_O; C, 74.81; H, 5.35; N, 16.16, Found: C, 75.02; H, 5.37; N, 16.22.

##### *(E)-1-(4-((1-Ethyl-[1,2,4]triazolo[4,3-a]quinoxalin-4-yl)amino)phenyl)-3-(4-methoxyphenyl)prop-2-en-1-one* (**7e**)

Yield (96%); m.p. 255–257 °C; IR cm^−1^ (KBr); 3438 (N–H), 2980 (C–H aromatic), 2921 (C–H aliphatic), 1731 (C=O), 1613 (C=N); ^1^H-NMR (DMSO-*d*_6_): 10.45 (br s, 1H, N–H exchangeable with D_2_O), 7.01–8.44 (m, 13H, Ar–H, 1H, C–H-arylidene), 3.82 (s, 3H, OCH_3_), 3.43 (q, 2H, *J* = 6.6, CH_3_CH_2_), 1.51 (t, 3H, *J* = 6.6, CH_3_CH_2_);^13^C-NMR (DMSO-*d*_6_) δ 189.7, 161.5, 159.4, 152.2, 146.7, 145.1, 142.1, 135.3, 132.2, 130.0, 128.5, 127.9, 127.5, 125.7, 124.8, 121.3, 120.4, 114.5, 112.8, 111.3, 55.6, 21.3, 12.1; MS (*m*/*z*), 450 (5.07, M + 1), 449 (18.27, M+), 420 (2.88), 316 (6.93), 265 (100), 252 (1.21), 237 (5.60), 212 (3.38), 184 (3.94), 128 (44.19), 77 (19.10); Anal. Calcd. for C_27_H_23_N_5_O_2_; C, 72.14; H, 5.16; N, 15.58, Found: C, 71.85; H, 5.18; N, 15.64.

##### *(E)-1-(4-((1-Ethyl-[1,2,4]triazolo[4,3-a]quinoxalin-4-yl)amino)phenyl)-3-(4-hydroxyphenyl)prop-2-en-1-one* (**7f**)

Yield (45%); m.p. 253–255 °C; IR cm^−1^ (KBr); 3482 (O–H), 3374 (N–H), 3120 (C–H aromatic), 2933 (C–H aliphatic), 1732 (C=O), 1662 (C=N); ^1^H-NMR (DMSO-*d*_6_): 10.45 (br s, 1H, N–H exchangeable with D_2_O), 9.02 (s, 1H, Ar–O–H exchangeable with D_2_O), 7.40–8.33 (m, 13H, Ar–H, 1H, C–H-arylidene), 3.44 (q, 2H, *J* = 7.2, CH_3_CH_2_), 2.36 (s, Ar*–*CH_3_), 1.46 (t, 3H, *J* = 7.2, CH_3_CH_2_); ^13^C-NMR (DMSO-*d*_6_) δ 189.7, 161.5, 157.4, 152.2, 146.7, 145.1, 142.1, 135.3, 132.2, 130.0, 128.5, 127.9, 127.5, 125.7, 124.8, 121.3, 120.4, 115.5, 112.8, 111.3, 21.3, 12.1; MS (*m*/*z*), 435 (5.59, M+), 330 (40.03), 316 (77.98), 261 (63.56), 257 (100), 97 (86.70), 81 (77.95); Anal. Calcd. for C_26_H_21_N_5_O_2_; C, 71.71; H, 4.86; N, 16.08, Found: C, 71.83; H, 4.87; N, 16.14.

##### *(E)-1-(4-((1-Ethyl-[1,2,4]triazolo[4,3-a]quinoxalin-4-yl)amino)phenyl)-3-(3,4,5-trimethoxyphenyl)prop-2-en-1-one* (**7g**)

Yield (11%); m.p. 247–249 °C; IR cm^−1^ (KBr); 3448 (N–H), 2981 (C–H aromatic), 2935 (C–H aliphatic), 1732 (C=O), 1653 (C=N); ^1^H-NMR (DMSO-*d*_6_): 10.48 (s, 1H, N–H exchangeable with D_2_O), 7.48–8.38 (m, 11H, Ar–H, 1H, C–H-arylidene), 3.43 (q, 2H, *J* = 7.2, CH_3_CH_2_), 2.56 (s, 9H, Ar*–*(OCH_3_)_3_), 1.51 (t, 3H, *J* = 7.2, CH_3_CH_2_); ^13^C-NMR (DMSO-*d*_6_) δ 189.4, 161.3, 153.4, 152.2, 146.7, 145.1, 142.1,138.4, 135.3, 132.2, 128.5, 127.9, 126.4, 125.7, 124.8, 121.5, 120.7, 112.4, 111.3, 103.5, 61.2, 56.3, 21.3, 12.1; MS (*m*/*z*), 510 (0.76, M + 1), 509 (1.16, M+), 330 (98.70), 261 (34.30), 233 (100), 212 (4.13), 206 (44.08), 158 (79.29), 128 (32.83), 77 (18.08); Anal. Calcd. for C_29_H_27_N_5_O_4_; C, 68.36; H, 5.34; N, 13.74, Found: C, 68.50; H, 5.35; N, 13.78.

##### *(E)-1-(4-((1-Ethyl-[1,2,4]triazolo[4,3-a]quinoxalin-4-yl)amino)phenyl)-3-(4-nitrophenyl)prop-2-en-1-one* (**7h**)

Yield (64%); m.p. 260–262 °C; IR cm^−1^ (KBr); 3368 (N–H), 3103 (C–H aromatic), 2935 (C–H aliphatic), 1794 (C=O), 1656 (C=N), 1534 (NO_2_); ^1^H-NMR (DMSO-*d*_6_): 10.49 (bs, 1H, N–H exchangeable with D_2_O), 7.42–8.39 (m, 13H, Ar–H, 1H, C–H-arylidene), 3.44 (q, 2H, *J* = 6, CH_3_CH_2_), 2.36 (s, Ar–CH_3_), 1.51 (t, 3H, *J* = 6, CH_3_CH_2_); ^13^C-NMR (DMSO-*d*_6_) δ 189.4, 161.3, 152.2, 147.4, 146.7, 145.1, 142.4, 141.1,135.3, 132.2, 129.3, 128.5, 127.9, 125.7, 124.8, 123.4, 121.5, 120.7, 112.4, 111.3, 21.3, 12.1; MS (*m*/*z*), 465 (0.72, M + 1), 464 (2.37, M+), 330 (47.53), 265 (31.53), 233 (47.84), 212 (2.54), 128 (16.32), 77 (32.13), 57 (73.05), 43.09 (100); Anal. Calcd. for C_26_H_20_N_6_O_3_; C, 67.23; H, 4.34; N, 18.09, Found: C, 67.04; H, 4.35; N, 18.16.

##### *(E)-3-(4-Chlorophenyl)-1-(4-((1-ethyl-[1,2,4]triazolo[4,3-a]quinoxalin-4-yl)amino)phenyl)prop-2-en-1-one* (**7i**)

Yield (73%); m.p. 255–257 °C; IR cm^−1^ (KBr); 3262 (N–H), 3091 (C–H aromatic), 2932 (C–H aliphatic), 1734 (C=O), 1656 (C=N), 1090 (C–Cl); ^1^H-NMR (DMSO-*d*_6_): 10.53 (s, 1H, N–H exchangeable with D_2_O), 7.38–8.68 (m, 13H, Ar–H, 1H, C–H-arylidene), 3.47 (q, 2H, *J* = 7.5, CH_3_CH_2_), 1.52 (t, 3H, *J* = 7.5, CH_3_CH_2_); ^13^C-NMR (DMSO-*d*_6_) δ 189.4, 161.3, 152.2, 146.7, 145.1, 142.4, 135.3, 133.5,133.2, 132.2, 129.3, 128.5, 127.9, 125.7, 124.8, 121.5, 120.7, 112.4, 111.3, 21.3, 12.1; MS (*m*/*z*), 455 (33.18, M + 2^)^, 454 (50.31, M + 1), 453 (100, M+), 316 (42.69), 233 (74.89), 181 (63.37), 137 (48.40), 128 (9.37), 102 (83.81), 77 (18.45); Anal. Calcd. for C_26_H_20_ClN_5_O; C, 68.80; H, 4.44; N, 15.43, Found: C, 68.76; H, 4.45; N, 15.49.

##### *(E)-3-(2,4-Dichlorophenyl)-1-(4-((1-ethyl-[1,2,4]triazolo[4,3-a]quinoxalin-4-yl)amino)phenyl)prop-2-en-1-one* (**7j**)

Yield (86%); m.p. 250*–*252 °C; IR cm^−1^ (KBr); 3441 (N–H), 2925 (C–H aliphatic), 1733 (C=O), 1682 (C=N), 1018 (C–Cl); ^1^H-NMR (DMSO-*d*_6_): 10.26 (bs, 1H, N–H exchangeable with D_2_O), 7.43–8.48 (m, 12H, Ar–H, 1H, C–H-arylidene), 3.49 (q, 2H, *J* = 6, CH_3_CH_2_), 1.46 (t, 3H, *J* = 6, CH_3_CH_2_); ^13^C-NMR (DMSO-*d*_6_) δ 189.4, 161.3, 152.2, 146.7, 145.1, 142.4, 136.7, 135.3, 132.2, 131.2, 130.3, 128.5, 128.1, 127.4, 126.7, 125.7, 125.2, 124.8, 121.5, 120.7, 112.4, 111.3, 21.3, 12.1; MS (*m*/*z*), 490 (7.29, M + 2), 489 (16.64, M + 2), 488 (21.86, M+), 487 (32.89), 316 (24.39), 265 (100), 233 (55.15), 212 (8.34), 171 (23.36), 128 (57.01), 90 (45.25), 77 (38.16), 40 (81.42); Anal. Calcd. for C_26_H_19_Cl_2_N_5_O; C, 63.94; H, 3.92; N, 14.34, Found: C, 63.85; H, 3.93; N, 14.39.

##### *(E)-3-(2,6-Dichlorophenyl)-1-(4-((1-ethyl-[1,2,4]triazolo[4,3-a]quinoxalin-4-yl)amino)phenyl)prop-2-en-1-one* (**7k**)

Yield (25%); m.p. 250–252 °C; IR cm^−1^ (KBr); 3384(N–H), 3070 (C–H aromatic), 2927 (C–H aliphatic), 1655 (C=O), 1597 (C=N), 1014 (C–Cl); ^1^H-NMR (DMSO-*d*_6_): 10.60 (s, 1H, N–H exchangeable with D_2_O), 7.46–8.39 (m, 12H, Ar–H, 1H, C–H-arylidene), 3.45 (q, 2H, *J* = 7.5, CH_3_CH_2_), 1.52 (t, 3H, *J* = 7.5, CH_3_CH_2_); ^13^C-NMR (DMSO-*d*_6_) δ 189.4, 161.3, 152.2, 146.7, 145.1, 142.4, 135.9, 135.3, 132.5, 132.2, 128.7, 128.0, 127.4, 126.7, 125.2, 124.8, 121.5, 120.7, 112.4, 111.3, 21.3, 12.1; MS (*m*/*z*), 490 (3.59, M + 1), 489 (9.96, M + 1), 488 (11.13, M+), 487 (16.43), 316 (75.45), 265 (8.27), 233 (100), 212 (13.18), 171 (24.14), 128 (29.10), 90 (89.94), 77 (99.74), 40 (79.75); Anal. Calcd. for C_26_H_19_Cl_2_N_5_O; C, 63.94; H, 3.92; N, 14.34, Found: C, 64.19; H, 3.93; N, 14.28.

### 4.3. Cancer Cell Antiproliferative Assay

The in vitro anticancer activities of the selected chalcone compounds against three cancer cell lines: human colon carcinoma (HCT-116), human hepatocellular carcinoma (HEPG-2), and human breast adenocarcinoma (MCF-7) were evaluated as described [56] with some modifications. Target tumor cells were grown to log phase in DMEM medium supplemented with 10% fetal bovine serum. After diluting to 1 × 10^5^ cells mL^−1^ with the medium, 100 μL of the obtained cell suspension was added to each well of 96-well culture plates. Subsequently, incubation was performed at 37 °C in 5% CO_2_ atmosphere for 48 h before the cytotoxicity assessment. Tested samples at preset concentrations were added to 6 wells with doxorubicin being employed as a positive reference. After 72 h exposure period, 25 μL of PBS containing 2.5 mg mL^−1^ of MTT was added to each well. After 4 h, the medium was replaced by 150 μL DMSO to dissolve the purple formazan crystals produced [57,58]. The absorbance at 570 nm of each well was measured with an ELISA plate reader. The data represented the mean of three independent experiments in triplicate and were expressed as means ± SD. The IC_50_ value was defined as the concentration at which 50% of the cells could survive from graphic plots.

### 4.4. EGFR Inhibition Assay

EGFR kinase activity was assessed using HTScan EGFR kinase assay kits (Cell Signaling Technology, Danvers, MA, USA). The experiments were performed according to the manufacturer’s instructions. In short, the GST-EGFR fusion protein was incubated with synthetic biotinylated peptide substrate and 10 μg/mL inhibitors in the presence of 400 μM ATP. Phosphorylated substrate was captured with strapavidin-coated 96-well plates. The level of phosphorylation was monitored by anti-phosphotyrosine and europium-labeled secondary antibodies (DELFIA, Perkin-Elmer, Akron, OH, USA). The enhancement solution was added at the end of the assay and enzyme activity was measured in a Victor II 1420 microplate reader (Wallac, Boston, MA, USA) at 615 nM.

### 4.5. Enzyme-Linked Immunosorbent Assay for Tubulin Beta (TUBb)

The HCT-116 cell line was obtained from the American Type Culture Collection (Manassas, VA, USA) and cultured using DMEM (Invitrogen/Life Technologies, Carlsbad, CA, USA) supplemented with 10% FBS (Hyclone, Logan, UT, USA), 10 mg/mL of insulin (Sigma, Mendota Heights, MN, USA), and 1% penicillin-streptomycin. Plate cells (cells density 1.2–1.8 × 10^3^ cells/well) in a volume of 100 mL complete growth medium and 100 mL of the tested compound per well in a 96-well plate for 18–24 h before the enzyme assay for tubulin. The microtiter plate provided in this kit has been pre-coated with an antibody specific to TUBb. Standards or samples are then added to the appropriate microtiter plate wells with a biotin-conjugated antibody specific to TUBb. Next, avidin conjugated to horseradish peroxidase (HRP) is added to each microplate well and incubated. After TMB substrate solution is added, only those wells that contain TUBb, biotin-conjugated antibody and enzyme-conjugated avidin will exhibit a change in color. The enzyme-substrate reaction is terminated by the addition of sulphuric acid solution and the color change is measured spectrophotometrically at a wavelength of 450 ± 10 nm. The concentration of TUBb in the samples is then determined by comparing the O.D. of the samples to the standard curve.

### 4.6. Molecular Docking

Molecular docking simulation was done for selected active target compounds into the three-dimensional complex of two biological targets including the crystal structures of EGFR (PDB code: 1M17) at 2.6 Å resolution and tubulin in complex with DAMA-colchicine and the stathmin-like domain (SLD) at 3.5 Å resolution (PDB: 3E22) [50] was carried out using the AutoDock software package (version 4.0) as implemented through the graphical user interface AutoDockTools (ADT) [59]. Prior to the calculations, crystallographic water and ligand molecules were removed from the X-ray structure. Hydrogen atoms were added to the structure with the Molecular Operating Environment (MOE, 2012) [60] and atomic partial charges were calculated using AutoDock Tools. Selected active chalcone compounds were docked into the active site of the targets to predict compound binding modes. For flexible docking, AutoDock standard parameter settings were applied. High-scoring binding poses were selected on the basis of visual inspection.

## 5. Conclusions

A novel series of chalcone derivatives with triazoloquinoxaline-linked moiety was designed, synthesized and evaluated as potent anticancer agents against a set of cancer cell lines. The structures of the novel compounds were confirmed using different spectroscopic techniques and elemental analyses. Different mechanistic investigations were done by testing the ability of the novel compounds to inhibit EGFR TK and tubulin polymerization. Most of compounds exhibited good potency against EGFR TK and tubulin at micromolar or submicromolar concentrations. An in-silico docking study was done to discover their binding modes within the targets pockets and a SAR screen was performed. Overall structural fragments contributed to the activity by different types of interactions like hydrogen bonding, hydrophobic, and aromatic stacking interactions. Taken together, a novel scaffold was introduced and its potent anticancer activity through dual inhibition of vital biological targets was revealed.

## Figures and Tables

**Table:** 

Compound	R1	R2	R3	R4	R5
**7a**	H	H	H	H	H
**7b**	OCH_3_	H	H	H	H
**7c**	OH	H	H	H	H
**7d**	H	H	CH_3_	H	H
**7e**	H	H	OCH_3_	H	H
**7f**	H	H	OH	H	H
**7g**	H	OCH_3_	OCH_3_	OCH_3_	H
**7h**	H	H	NO_2_	H	H
**7i**	H	H	Cl	H	H
**7j**	Cl	H	Cl	H	H
**7k**	Cl	H	H	H	Cl

**Table 1:** Cytotoxicity screening of selected chalcone derivatives against three different cancer cell lines.

Compounds	IC_50_ (µM) *		
	HCT-116	MCF-7	HepG-2
**5**	236 ± 0.10	246 ± 0.13	322 ± 0.15
**6**	411 ± 0.21	410 ± 0.25	446 ± 0.20
**7a**	57.49 ± 0.29	58.92 ± 0.32	105.21 ± 0.50
**7b**	25.60 ± 0.03	24.04 ± 0.12	30.72 ± 0.12
**7c**	19.76 ± 0.23	22.17 ± 0.11	17.32 ± 0.12
**7d**	>100	93.40 ± 0.14	>100
**7e**	9.57 ± 0.11	8.23 ± 0.25	34.28 ± 0.14
**7f**	18.10 ± 0.15	>100	>100
**7g**	3.61 ± 0.18	1.65 ± 0.13	8.58 ± 0.06
**7h**	>100	>100	>100
**7i**	>100	>100	>100
**7j**	>100	>100	>100
**7k**	19.66 ± 0.25	>100	>100
Doxorubicin	1.55 ± 0.03	0.27 ± 0.08	0.22 ± 0.01

* Cytotoxic effects of selected chalcone compounds were reported on colon, breast, and liver cell lines following exposure to different concentrations of compounds, and cell viability was assessed using the MTT method.

**Table 2:** Inhibitory data of the selected compounds to both EGFR TK and tubulin polymerization.

Compounds	EGFR Inhibition (IC_50_ µM) ^a,b^	Tubulin Polymerization (IC_50_ µM) ^c^
**7a**	661.0 ± 0.11	77.1 ± 0.21
**7b**	127.0 ± 0.32	18.1 ± 0.30
**7c**	136.0 ± 0.05	43.4 ± 0.01
**7d**	>100	ND
**7e**	0.083 ± 0.04	14.7 ± 0.11
**7f**	>50	>100
**7g**	0.039 ± 0.16	8.84 ± 0.02
**7h**	>100	>100
**7i**	>100	>100
**7j**	>100	>100
**7k**	>100	>100
Colchicine	-	26.8 ± 0.12
Staurosporine	0.054 ± 0.10	-

^a^ Values are averages of three independent experiments; ^b^ Compounds tested at concentration of 10 μM; ^c^ Values are averages of two independent experiments.

**Table 3:** Docking scores, amino acid interactions and interacting groups of the docked compounds into the active site of EGFR-TK and tubulin.

Compound	Target	Binding Energy (kcal/mol)	Interacting Moieties	Amino Acids
**7e**	EGFR TK (1M17)	−13.51	3,4 diO-CH_3_	Lys721
			C=O iminone linker	Met769
			Quinoxaline	Lys704
			4-aminophenylcarbonyl	Leu694
**7g**		−15.5	3,4 diO-CH_3_	Lys721
			C=O iminone linker	Met769
			Quinoxaline	Lys704, Pro770, Leu768
			4-aminophenylcarbonyl	Leu694
	Tubulin (3E22)	−12.8	3,4 diO-CH_3_	Cys241, Thr240
			Quinoxaline	Thr179
			Chalcone	Leu248, Ala315, Ile378, Leu255
